# Teams with lower injury rates have greater success in the Currie Cup rugby union competition

**DOI:** 10.17159/2078-516X/2019/v31i1a6401

**Published:** 2019-01-01

**Authors:** L T Starling

**Affiliations:** Division of Exercise Science and Sports Medicine, Department of Human Biology, University of Cape Town, Cape Town, South Africa

**Keywords:** rugby, performance, injury prevention, health education

## Abstract

**Background:**

Professional football teams that rank high on the log at the end of the season generally have fewer injuries than teams that rank lower on the log. This highlights the importance of implementing injury prevention measures, not only to protect player welfare and ensure their longevity in the sport, but also to improve the performance of the team. The association between a low incidence of injury and superior performance during a season may be even more relevant in sports with a higher incidence of injury than football, such as rugby union.

**Discussion:**

To examine this association in the South African Currie Cup rugby union competition, time-loss (≥ 1 day training/match play missed) injury data and final position in the competition was examined over five-seasons. Teams who ranked in 1^st^ position had significantly lower average injury rates than teams who ranked in last position [48 injuries per 1 000 player hours (95% C.I 20 to 76) vs 130 injuries per 1 000 player hours (95% C.I 79 to 180)]. More specifically, the team with the lowest injury rate in each season ranked in 1^st^ or 2^nd^ position. This team performance aspect of injury prevention should be highlighted more. In particular, this should be used to assist with communicating the importance of injury prevention programmes to stakeholders directly involved with budgetary allocations in the team.

Success in team sports is the result of the effective combination of numerous factors, such as physical fitness, psychological factors, tactical strategies and player skill level.^[1]^ The influence of injuries on team success is a factor which is under-represented in the literature. Injuries result in players being unavailable for selection which, depending on the severity of the injury, may fluctuate from one match to the whole season. This may hamper the selection of the strongest team or force the coach to use different player combinations. Both of these points can potentially result in changes to team strategy, disruption of team dynamics and invoke psychological stress and anxiety among teammates during match preparation. It has also been found that pre-injured athletes often experience heightened anxiety when re-entering the team due to a lack of confidence in the previously injured body part and uncertainty around their return to the pre-injury level of performance.^[2]^ An athlete experiencing re-injury anxiety may be hesitant to push at maximum effort which, in conjunction with their affected psychological state, may negatively affect team dynamics and performance.^[2]^

Studies in professional football have reported strong correlations between lower injury measures and team success.^[6–8]^ The association between a low incidence of injury and superior performance during a season may be even more relevant in sports with a higher incidence of injury than football, such as rugby union. To date only two studies have examined the association between injuries and team success in rugby union.^[3,4]^ The first study showed a moderate correlation between average days lost per team and final league position across two seasons in professional teams.^[3]^ The other study reported negative associations between injury measures in elite players and team success across seven seasons.^[4]^ It is tempting to conclude an association between injuries and team success, however, more data are needed before this can be made with any confidence. This is a challenge to examine in a study because many factors influence team success which have to be accounted for. For example, changes in coaching and medical staff across seasons, the relevant importance of each player in the squad, and changes in competition structure across the years would require data to be collected over several seasons to provide sufficient statistical power for this analysis. An opportunity to investigate this question in the Currie Cup competition, the South African Rugby Union Premiership Division competition, arose through the SA Rugby Injury and Illness Surveillance and Prevention Project, which is entering its sixth year.^[5]^

## Discussion

### Time-loss injuries and team success in the Currie Cup competition

**Fig. 1:**
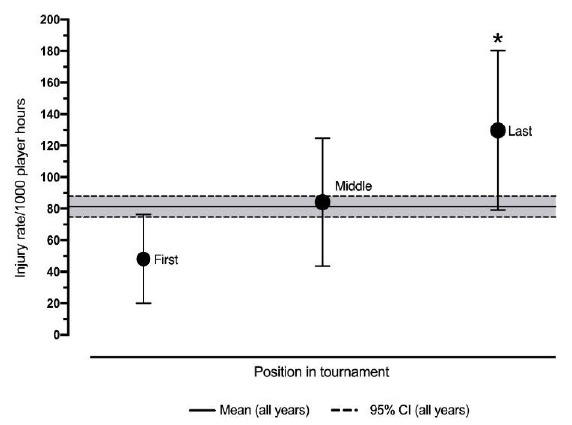
Average injury rate/1 000 player hours (95% CI) for time-loss match injuries for teams ranked in first (n = 5), middle (n = 5) and last (n = 5) position in the Currie Cup competition from 2014 – 2018. Asterisks (*) indicates average injury incidence is significantly different to another group.

When considering the average time-loss (≥ 1 day training/match play missed) injury rate of teams who ranked at the top, in the middle, and at the bottom of the competition across five seasons of the Currie Cup, there was an increase in the average injury rate for teams which ranked further away from first position. The average injury rate of teams in first position was significantly lower than those in last position [48 injuries per 1 000 player hours (95% C.I 20 to 76) vs 130 injuries per 1 000 player hours (95% C.I 79 to 180)] (Fig. 1). This trend, whereby teams who are more successful in the competition have lower injury rates than those who are less successful, follows the pattern found in both international rugby union ^[3,4]^ and football data respectively.^[6–8]^

It is apparent that across five seasons of the Currie Cup competition the teams in first position had, on average, a significantly lower injury rate than those in last position. Table 1 presents the final competition position of each team for each year, with each letter representing a team in the competition. The team with the lowest time-loss injury rate for each year is highlighted in yellow. Looking at the final competition position of the team with the lowest injury rate for each year, these teams are either in first or second position (Table 1).

Epidemiological studies identify injury risk factors and areas where sports governing bodies can implement injury prevention strategies.^[5]^ However, successful implementation of an injury prevention measure requires support from all key stakeholders. Injury prevention strategies are often communicated through the importance of protecting player welfare, with the need for the intervention justified by how it will reduce the risk of injury in a certain aspect of the game. Injury prevention strategies communicated in this respect generally appeal to stakeholders whose primary interest is in the medical management of players. The importance of an injury prevention measure may be better explained to other stakeholders in a team through assessing the association between injuries and the primary interest of that stakeholder. For coaches, whose primary interest is in the performance of the team, the influence of injuries on performance should be explored. Coaches are more likely to be concerned about factors which contribute to team success and which can be controlled, such as player fitness levels and tactical strategies. It may be more practically relevant to coaches to describe the interplay between injuries, these factors and performance. Administrators with budgetary control also need to be aware of the influence of injuries on performance to highlight the importance of allocating funds to injury prevention resources. There are many confounding factors which need to be considered when inferring an association between injuries and performance, however, to encourage an inclusive decision and buy-in from all stakeholders in a team. The interplay between injuries, performance and budget should also be explored. There needs to be a paradigm shift in the communication of injury prevention strategies to these key stakeholders, whose primary interest is in the performance of the team.

## Conclusion

Teams who perform better in the Currie Cup competition have lower injury rates than those who perform poorly. This association should be used to highlight the importance of injury prevention to key stakeholders of teams. This communication approach to coaches and administrators may improve their support and adoption of comprehensive injury prevention programmes.

## Figures and Tables

**Table 1:** Final position of each team in the Currie Cup competition from 2014 – 2018, with each letter representing a team in the competition. The team with the lowest injury rate in each year is highlighted in yellow.

Final competition position	2014	2015	2016	2017	2018
**1** **^st^**	A	**B**	E	**A**	**C**
**2** **^nd^**	**B**	A	**D**	C	A
**3** **^rd^**	C	D	A	B	B
**4** **^th^**	D	E	B	D	D
**5** **^th^**	E	C	C	E	F
**6** **^th^**	F	F	H	F	H
**7** **^th^**	G	G	I	H	E
**8** **^th^**	H	H	F		
**9** **^th^**			G		
